# Application of New *Yarrowia lipolytica* Transformants in Production of Citrates and Erythritol from Glycerol

**DOI:** 10.3390/ijms25031475

**Published:** 2024-01-25

**Authors:** Anita Rywińska, Ludwika Tomaszewska-Hetman, Zbigniew Lazar, Piotr Juszczyk, Patrycja Sałata, Karolina Malek, Adrian Kawecki, Waldemar Rymowicz

**Affiliations:** Department of Biotechnology and Food Microbiology, The Faculty of Biotechnology and Food Science, Wroclaw University of Environmental and Life Sciences, Chełmońskiego Str. 37, 51-630 Wrocław, Poland; anita.rywinska@upwr.edu.pl (A.R.); zbigniew.lazar@upwr.edu.pl (Z.L.); piotr.juszczyk@upwr.edu.pl (P.J.); patrycja.salataa@gmail.com (P.S.); 121139@student.upwr.edu.pl (A.K.); waldemar.rymowicz@upwr.edu.pl (W.R.)

**Keywords:** *Yarrowia lipolytica*, glycerol, citrates, erythritol, genetic engineering

## Abstract

Citric acid and erythritol are obtained on an industrial scale using biotechnological methods. Due to the growing market demand for these products, research is underway to improve the process economics by introducing new microorganisms, in particular of the species *Yarrowia lipolytica*. The aim of this study was to evaluate transformants of *Y. lipolytica* for growth and ability to overproduce citric acids and erythritol from glycerol. The transformants were constructed by overexpressing glycerol kinase, methylcitrate synthase and mitochondrial succinate-fumarate transporter in the mutant Wratislavia 1.31. Next, strains were assessed for biosynthesis of citrate (pH 5.5; nitrogen limitation) and erythritol (pH 3.0; high osmotic pressure) from glycerol. Regardless of culture conditions strains, 1.31.GUT1/6 and 1.31.GUT1/6.CIT1/3 exhibited high rates of substrate utilization. Under conditions favoring citrate biosynthesis, both strains produced several percent more citrates, accompanied by higher erythritol production compared to the parental strain. During erythritol biosynthesis, the strain 1.31.GUT1/6.CIT1/3.E34672g obtained as a result of co-expression of all three genes stood out, producing 84.0 g/L of erythritol with yield and productivity of 0.54 g/g and 0.72 g/Lh, respectively, which places it in the group of the highest-ranked producers of erythritol among *Y. lipolytica* species.

## 1. Introduction

*Yarrowia lipolytica* is a species of unconventional yeast whose specific properties have attracted great interest among scientists and industry [1]. This species can be found in food products rich in proteins and fats [2] as well as in soil, oil-contaminated areas and marine environments [3]. Occurrence in such diverse ecosystems is related to the abundance of substrates that they can assimilate. Previous studies have shown that this yeast assimilates monosaccharides, free fatty acids and a wide range of long-chain hydrocarbons [4,5]. *Y. lipolytica* is characterized by an efficient mechanism of uptake of hydrophobic substrates and their efficient metabolism, which consequently leads to lipid storage [6,7,8]. Due to their high lipid accumulation potential, this species has become a model organism, accumulating more than 20% of biomass in the form of triacylglycerides and sterol esters, which may be a promising alternative to commonly used fuels [9,10].

The studies on *Y. lipolytica* metabolism have confirmed that the assimilated substrate has a direct impact on the type of metabolite produced, as these yeast, in the presence of hydrophilic substrates or their mixtures, efficiently produce organic acids [11,12] and polyols [13,14] as well as engineered strains, e.g., carotenoids [15,16,17] and terpenes [18,19]. The direction and efficiency of metabolites’ biosynthesis are also influenced by culture parameters. The dynamic progress of biological sciences has generated knowledge confirming correlations of secreted compounds in response to abiotic environmental factors. As is known, osmotic stress induced by high concentrations of sugar, mineral salts or ethanol leads to an uncontrolled decrease in cell turgor and activation of a cascade of defense mechanisms resulting in erythritol production and accumulation [20]. In yeast cells, erythritol is produced as an osmoprotective agent in the pentose-phosphate pathway, where the final step is catalyzed by erythrose reductase, which reduces erythrose to erythritol with simultaneous oxidation of NAD(P)H [21,22]. Erythritol, increasingly considered the best substitute for white sugar, is of great interest as an alternative non-caloric sweetener, especially among populations struggling with overweight diabetes or autoimmune diseases. This sugar alcohol, designated the E number E968, does not cause a rise in blood insulin level, making it safe to consume even for those suffering from metabolic syndrome [23,24].

Equally interesting is the response of *Y. lipolytica* yeast to stress induced by a deficit of one of the nutrients. Previous studies have shown that nitrogen limitation inhibits the growth phase, disrupts the Krebs cycle and shifts the relevance of metabolic reactions towards the synthesis of organic acids, especially citric acid [25,26]. This is closely related to the blockade of anaplerotic reactions requiring intermediates of this amphibolic pathway, the increased supply of acetyl-CoA and oxaloacetate precursors, and the relatively low rate of citrate catabolism. Citric acid is described as a non-hazardous compound and, as such, has found applications in the pharmaceutical, food, chemical, biomedical and environmental industries. Its widespread use has led to its classification as safe and assignment of the E numbers E330 for citric acid, E331 for sodium citrate and E332 for potassium citrate [27]. Most of all, it is one of the most popular preservatives in the world—as a natural antioxidant, it prevents premature spoilage of food and additionally inhibits color change. It is used, for example, in commercial egg products to prevent color loss during cooking, and furthermore, it is thought to improve the foaming and emulsifying properties of proteins [28]. Citric acid is also used as a crosslinking agent, correcting pH and chelating metal ions in antioxidant systems [29]. Its application can serve as an inhibitor of kimchi browning [30]. It is worth noting that citric acid is also used as a crosslinking compound in the production of biodegradable plastics such as polyvinyl alcohol/starch [31], polyvinyl alcohol/xylan [32], starch films [33] and tragacanth gums [34]. According to a recent market report, the global citric acid market reached a volume of 2.39 million tons in 2020 and is further expected to reach 2.91 million tons by 2026 [35].

Both citric acid and erythritol are produced on an industrial scale by biotechnological methods. For decades, global demand for citric acid has been met through biosynthesis using filamentous fungi, with only about 1% being obtained from lemons. In turn, industrial erythritol production uses *Moniliella pollinis*, *M. megachiliensis* and *Y. lipolytica*. Currently the SN-G42 strain of *M. megachiliensis*—the mutant strain of SN-124A—showed the best production parameters with a small amount of byproducts [36]. The implementation of new strains of *Y. lipolytica* for erythritol biosynthesis is gaining popularity, not least because of the wide range of substrates which may be applied for its production. The use of the most abundant substrates, especially waste products such as glycerol, pomace and paraffins is becoming the basis for increasing the economic justification of the process. Glycerol, as a waste generated in biodiesel production, is one of the substrates whose ever-increasing surplus is becoming a problem to manage and *Y. lipolytica* is a very promising microorganism able to efficiently utilize this byproduct [37,38]. Recently, new studies have been published dealing with the optimization of processes for the production of citrate and erythritol from glycerol and the search for new strains with higher product titers [39]. As is well known, a good strategy for improving the properties of microorganisms is mutagenesis of pre-selected strains; nevertheless, this is a time-consuming and labor-intensive solution [40]. Given the available completely sequenced genome of *Y. lipolytica*, it became possible to identify and analyze genes at the molecular level [41]. Understanding the regulation of biochemical synthesis pathways may help to achieve higher productivity or metabolite yield [24,42] because it indicates crucial points in the cell metabolism, at the stage of substrate utilization, synthesis and secretion of specific metabolites, that can be controlled and regulated on the one hand by adjustment of culture conditions, and on the other by targeted genome engineering. Studies on citric acid and erythritol production confirmed the positive effect of the overexpression of genes encoding the enzymes responsible for glycerol utilization [43]. The consequences of overexpression of the *CIT1* gene encoding mitochondrial citrate synthase involved in citrate biosynthesis were investigated as well [44]. However, the influence of this modification on the process of biosynthesis of erythritol from glycerol has not been assessed so far. Similarly, the effect of overexpression of the *YALI0E34672g* gene, encoding a mitochondrial organic acid transporter, on the biosynthesis of citrate and erythritol has not yet been investigated. Moreover, it seems interesting to assess the possible synergism of the overexpression of genes controlling various points along the biosynthetic pathway of these compounds. Therefore, the aim of this study was to evaluate *Y. lipolytica* Wratislavia 1.31 and its transformants overexpressing one or a combination of two or three genes of *GUT1*, *CIT1*, or *YALI0E34672g* for growth and the ability to overproduce citric acid and erythritol from glycerol.

## 2. Results and Discussion

The methodology used for engineering the metabolism of *Y. lipolytica* enables the integration of expression cassettes into a random locus in the yeast genome because of the retrotransposon sequences (ZETA sequences). Thus, additional and random phenotype changes might appear in the engineered cell. Due to that, the basic characteristics of the obtained transformants, such as growth and colony morphology on solid media, as well as growth kinetics in liquid medium, were examined. In addition, it was verified whether the introduced modifications altered the nutritional requirements of the analyzed transformants by accidentally deleting some of the key genes related to the functioning of basic metabolic pathways.

### 2.1. Evaluation of Yeast Growth on Glycerol Using Drop-Test Analysis

**Figure 1:**
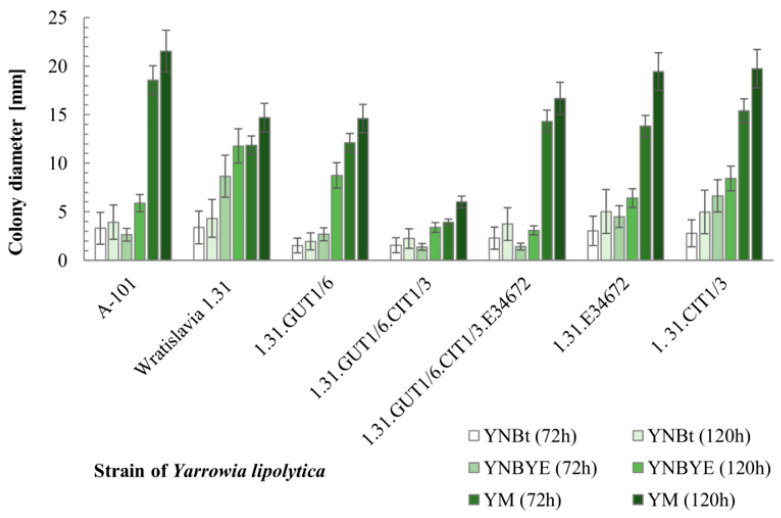
Colony diameter of *Y. lipolytica* strains growing on minimal yeast nitrogen base (YNB) and rich yeast extract (YE) medium. YNB_t_—thiamine (6 µg/L) enriched medium; YNB_YE_—yeast extract (0.1 g/L) enriched medium. The results presented are the mean values of three independent biological replications.

The morphology and colony size of seven strains of *Y. lipolytica* were evaluated. Among the yeasts being tested, the wild type strain A-101 and its acetate-negative mutant Wratislavia 1.31, i.e., the parental strains for the obtained transformants, were included. The transformants were constructed by overexpressing *GUT1* (glycerol kinase), *CIT1* (methylcitrate synthase) and the *YALI0E34672g* gene, encoding the mitochondrial succinate-fumarate transporter. Transformants contained multiple copies of single genes or their combinations—strains with two (CIT1.GUT1) or three genes (CIT1.GUT1.E34672g)—were obtained. Thiamine (YNB_t_) and yeast extract (YNB_YE_) enriched minimal medium, as well as rich growth medium (YM) were used for growth evaluation. All strains were able to grow on the tested media (Figure 1), and their colonies were cream-colored, exhibiting no differences in morphology.

The largest colonies were observed on YM medium, and the smallest on YNB_t_. On YNB_t_ medium, the largest colony diameter was observed for strains 1.31.CIT1/3 and 1.31.E34672g. On this medium, significantly smaller colonies, compared to the parental strain, were found for strains 1.31.GUT1/6 and 1.31.GUT1/6.CIT1/3. On YNB_YE_ medium, the best growing strain was Wratislavia 1.31 and among the transformants analyzed, 1.31.GUT1/6 and 1.31.CIT1/3. In contrast, the smallest colonies on YNB_YE_ were observed for strains 1.31.GUT1/6.CIT1/3 and 1.31.GUT1/6.CIT1/3.E34672g. On YM medium after 120 h, the largest colony diameter of 21.54 mm was noted for the parental strain A-101, and among the transformants, strains containing one gene: 1.31.E34672g and 1.31.CIT1/3. By far the smallest colonies on YM medium were observed for strain 1.31.GUT1/6.CIT1/3. The discussed growth ability differences may indicate that some of the expression cassettes were inserted into loci of the genome containing some important genes, however, the insertion site was not analyzed in this study.

### 2.2. Evaluation of Yeast Growth Kinetics in the Presence of Glycerol by Microculture Method

**Figure 2:**
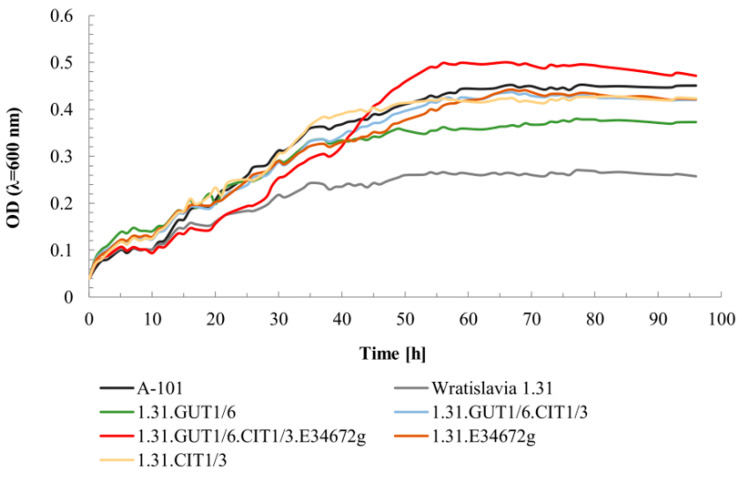
Growth curves of *Y. lipolytica* strains cultivated on mineral medium with thiamine deficiency. Quintuple experiments were performed at 28 °C under constant agitation using Spark Tecan apparatus. For abbreviations see the Abbreviations section of this document.

In the next stage of the study, the growth parameters of the newly obtained *Y. lipolytica* transformants were compared to those of the parental strains, A-101 and Wratislavia 1.31. The cultures were carried out in a Spark Tecan device in medium containing 20 g/L glycerol and the growth limiting factor was a thiamine concentration of 3 μg/L. The growth kinetics are shown in Figure 2.

All analyzed strains were characterized by a similar time of adaptation to environmental conditions (the lag phase lasted about 10 h). Generally, transformants and the A-101 strain reached the stationary phase later than Wratislavia 1.31 but at a higher value of OD_600_ (Figure 2, Table 1). Interestingly, the final level of OD_600_ was highest for strain 1.31.GUT1/6.CIT1/3.E34672g, which also stood out in the previous experiment due to its colony size on YNBt-agar minimal medium. This observation was quite surprising, as this transformant needs to spend the highest amount of energy in the production of all three overexpressed proteins, but its genotype seems to favor its growth anyway. The integration sites of the cassettes for gene overexpression will have to be investigated. The highest µ_max_ value, 0.1 h^−1^, was obtained by the wild-type A-101 strain, as well as 1.31.CIT1/3 and 1.31.E34672g strains (Table 1), which, in turn, stood out for their large colonies on YM-agar medium (Figure 1).

### 2.3. Citrates Biosynthesis

**Figure 3:**
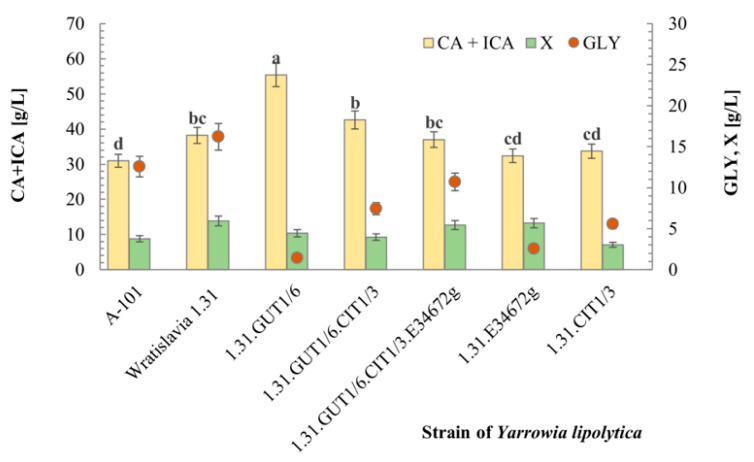
Characterization of citrate biosynthesis by wild-type strain A-101, the acetate-negative mutant Wratislavia 1.31 and transformants of *Y. lipolytica* in shake flask cultures. Values marked with different letters differ significantly at *p* ≤ 0.05. For abbreviations see the Abbreviations section of this document.

A comparison of strains in terms of citrate production from glycerol was carried out in shake flask cultures in medium with 100 g/L of glycerol, where yeast growth was limited by the concentration of the nitrogen source (1 g/L of NH_4_Cl). After 168 h of cultivation, the residual glycerol concentration in the post-culture medium ranged from 1.4 (strain GUT.1/6) to 16.2 g/L (strain Wratislavia 1.31), as shown in Figure 3. Strains with an additional copy of one gene— *GUT1*, *CIT1* and *YALI0E34672g*—showed a higher rate of substrate utilization. The positive effect of *GUT1* gene overexpression on the rate of glycerol utilization by *Y. lipolytica* has already been observed in other studies [40,43]. However, in our study, the sum of citrates secreted to the medium was highest for the 1.31.GUT1/6 strain (Figure 3). The *GUT1* gene encodes glycerol kinase, an enzyme that phosphorylates glycerol to 3-phosphoglycerol, which in turn is converted to phosphodihydroxyacetone through the action of glycerol-3-P dehydrogenase, which is further used in glycolysis [45,46]. Univariate analysis of variance distinguished five homogeneous groups, and the strain GUT.1/6 was included in the first distinct group (a), which statistically distinguishes it as a particularly good citrate producer (Figure 3). A slight improvement in citrate production was also found in the strain containing 2 genes, *GUT1* and *CIT1*, which was also assigned to a separate group (b). On the other hand, it was surprising that there was no positive effect of *CIT1* gene overexpression due to its citrate synthase activity [44], but it is worth noting that only in the culture of strain 1.31.CIT1/3 were no other products than citric acids observed. In the case of the other strains, the amount of polyols, pyruvic acid and α-ketoglutaric acid in the total products ranged from 0.9 to 3.6%.

Various modifications of the metabolism of *Y. lipolytica* were applied to improve citrate biosynthesis and different substrates were tested as carbon sources. The results from different studies and scientific groups are summarized in Table 2. Genetic engineering of the A18 strain, a derivative of the A-101 wild-type strain, was carried out by Celińska and Grajek [47]. The modifications proposed by the authors contained three heterologous genes (*dhaB1*, *dhaB2* and *dhaT*). The genes *dhaB1* and *dhaB2* obtained from *Clostridium butyricum* encode a vitamin B_12_-independent glycerol dehydratase and its reactivator.

The *dhaT* gene encodes a wide-spectrum alcohol oxidoreductase and was obtained from *Shimwellia blattae* (formerly *Escherichia blattae*). The obtained recombinant strain NCYC3825 produced 58.8 g/L of citrate compared to 10.4 g/L obtained in the same conditions by its parental strain, however, the modifications introduced primarily caused high biomass and lipids accumulation. Förster et al. [52] focused on improving the citric/isocitric acid ratio (CA/ICA) by constructing strains with increased isocitrate lyase activity. In fact, the selective overexpression of the *ICL1* gene in the multicopy transformants minimized the secretion of the unwanted by-product, isocitrate from 10–12% to 3–6% in glucose, sucrose or glycerol media. After expression of the *PYC1* gene (encoding pyruvate carboxylase) from the marine fungus *Penicillium rubens* 1607 in the SWJ-1b strain of *Y. lipolytica*, the authors obtained five strains that in shake flask cultures produced more citrate than the parental strain. In other studies, the analyzed transformant PR32 showed much higher citric acid production, 70.2 g/L, compared to 27.3 g/L secreted by its parental strain SWJ-1b from 120 g/L of glucose at 144 h [50]. Furthermore, expression of the *PYC1* gene from *Meyerozyma guilliermondii* in *Y. lipolytica* SWJ-1b resulted in a twofold increase in citric acid production—obtained by the PG86 transformant [49]. Strains engineered to utilize inulin by expressing the *Kluyveromyces marxianus INU1* gene in the *Y. lipolytica* Wratislavia K1 strain (transformant K1 INU 6) and *Y. lipolytica* AWG7 strain (transformant AWG7 INU 8) showed approximately 10% and 55% higher citric acid production from inulin compared to citric acid biosynthesis from the inulin building block—fructose, respectively [39,48]. In contrast, in the present study, glycerol was used as a substrate and modification of yeast to improve assimilation of this compound, which showed the most beneficial effect for citrate biosynthesis. The introduction of additional copies of the *GUT1* gene into the strain Wratislavia 1.31, which is an acetate-negative mutant of the A-101 strain, resulted in a 1.4-fold increase in production (compared to the control strain) under shake flask culture conditions. Strains 1.31.GUT1/6 and 1.31.GUT1/6.CIT1/3 with additional copies of the *GUT1* and *CIT1* genes were also examined for citric acid biosynthesis in batch cultures in a bioreactor. For comparison, cultures involving strains A-101 and Wratislavia 1.31 were also performed. The citric acid biosynthesis process was carried out using 150 g/L of glycerol as the sole carbon source in a stirred tank bioreactor in a 2 L working volume. The obtained results of these cultures are summarized in Table 3. The total substrate depletion was observed to be fastest in the culture of the 1.31.GUT1/6.CIT1/3 transformant and at the same time the final biomass concentration of this strain was the highest among the analyzed strains. In contrast, the highest sum of citric acids secreted to the medium was obtained for the strain 1.31.GUT1/6. Unfortunately, the proportion of isocitric acid in the total citric acids produced was as high as 31%. Such a high amount of isocitric acid was similar to the proportion of citric/isocitric acid produced by wild-type strain A-101, and indicated that the overexpression of the *GUT1* gene led to improved glycerol utilization which induced the Krebs cycle, finally resulting in a significant increase in the production of this unwanted metabolite when compared to the strain Wratislavia 1.31. However, a similar modification introduced in the genome of the wild strain A-101 resulted in much better improvement of citric acid production parameters, especially at pH = 3.0 [43]. The AJDpADUTGut1 strain produced 60.4 g/L citric acid in 96 h, while the parental strain secreted only 4.4 g/L at this low pH. The authors obtained even better results using the AJDpADUT-Gut1/2 strain obtained by co-expression of the *YALI0F00484g* gene (*GUT1*) encoding glycerol kinase and the gene *YALI0B02948g* (*GDH*) encoding glycerol-3-P dehydrogenase. However, analysis of the citric/isocitric acid composition showed that isocitric acid constituted 56% of total citric acids [51], which in this case can support the observations obtained in our studies. Furthermore, Hapeta et al. (2020) [44] constructed two transformants of *Y. lipolytica* Wratislavia 1.31 overexpressing the *CIT1* or *CIT2* gene, encoding 2-methyl citrate synthase and citrate synthase, respectively. It was very interesting that in both strains an increased contribution of isocitric acid to the total citric acids pool was observed. This study further confirms the results obtained in our study showing that pushing the carbon flux through the citric acid cycle results in higher isocitric acid biosynthesis and secretion. However, in addition to the increased proportion of isocitric acid, in the cultures of both transformants analyzed in the present work, there was also a surprisingly high proportion of erythritol in the total amount of products obtained, which indicated relatively low selectivity of isocitric acid production by the analyzed transformants (Table 3). The formation of polyols under growth-limiting conditions—caused by nitrogen limitation, which is a prerequisite parameter for citrate overproduction—has been previously observed, although the mechanism of this process is not well understood [53]. Certainly, osmotic pressure caused by high concentrations of the substrate and by the growing concentration of the resulting product is one of the factors responsible for this phenomenon. This theory is supported by higher concentrations of erythritol determined in cultures with glycerol than with the same concentrations of glucose, because glycerol generates higher osmotic pressure [53]. However, increased concentration of erythritol in the post-culture broth was also observed in glucose-based medium for *Y. lipolytica* transformants overexpressing hexokinase and hexose transporters [54]. This observation supports the theory that higher carbon flux through the cells’ metabolism favors the formation of a protective metabolite such as erythritol.

Another factor favoring overproduction of erythritol by *Y. lipolytica* is the low pH of the culture. For comparison, the Wratislavia 1.31 strain produced only 4.8 g/L erythritol at pH 5.5, which was intended as optimal for citric acid biosynthesis, while it produced 26.2 g/L erythritol when cultured at pH 3.0 [21]. This concentration was very close to that obtained in the present study for strains with additional copies of *GUT1*; however, it should be noted that cultures were conducted at a pH optimal for citric acid biosynthesis.

### 2.4. Biosynthesis of Erythritol

Previous studies on the biosynthesis of erythritol from glycerol by *Y. lipolytica* indicated that efficient overproduction of this metabolite requires an acidic environment and elevated osmotic pressure. Such conditions were used in the present study for characterization of the obtained transformants—cultures were conducted at pH 3.0 in a medium containing 150 g/L of glycerol and 25 g/L of NaCl. The generated osmotic pressure reached 2.9 Osm/kg at the beginning of the culture. Previous studies have also confirmed that the erythritol biosynthesis pathway requires activity of enzymes responsible for glycerol utilization, glycerol kinase and glycerol-3-P dehydrogenase, as well as enzymes from the pentose-phosphate pathway [21]. Until now, overexpression of one or both genes of glycerol metabolism, glycerol kinase and glycerol-3-P dehydrogenase, has been analyzed to improve erythritol production by *Y. lipolytica* [43,55]. In fact, the authors observed a positive effect on erythritol biosynthesis over the parental strains only for overexpression of the *GUT1* gene (encoding glycerol kinase) or of both *GUT1* and *GUT2* (encoding glycerol-3-P dehydrogenase) genes, while overexpression of *GUT2* alone had no effect on the dynamics of erythritol production or the glycerol consumption rate.

**Figure 4:**
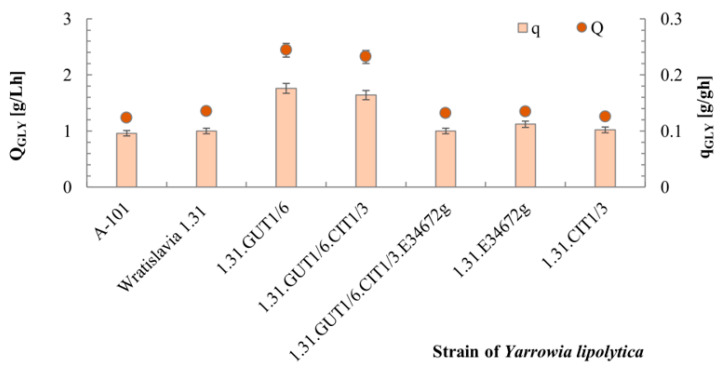
Dynamics of glycerol utilization under erythritol biosynthesis conditions by different *Y. lipolytica* strains during bioreactor cultures with glycerol. For abbreviations see the Abbreviations Section of this document.

Among the yeasts tested in this study, the strain with an additional copy of the *GUT1* gene (1.31.GUT1/6) utilized glycerol most rapidly under erythritol biosynthesis conditions (Figure 4). The volumetric (Q_GLY_) and specific (q_GLY_) rates of glycerol consumption were 2.44 g/Lh and 0.176 g/gh, respectively. However, high values of the q_GLY_ and Q_GLY_ parameters were also observed for the strain with overexpression of the two genes *GUT1* and *CIT1* (1.31.GUT1/6.CIT1/3).

**Figure 5:**
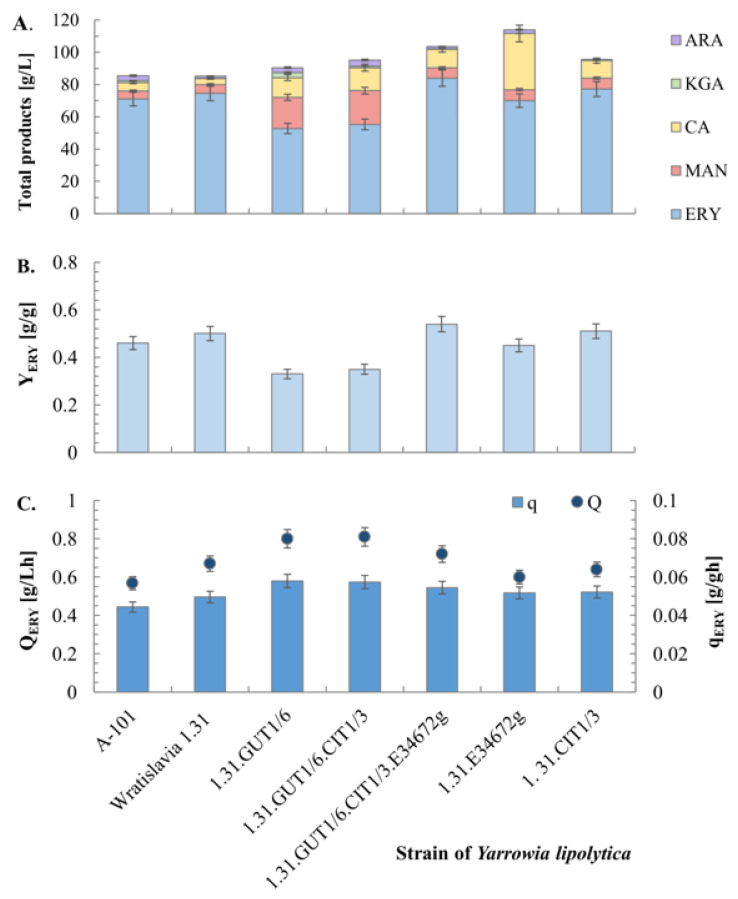
Concentration of erythritol and by-products (**A**), erythritol yield (**B**) and dynamics (**C**) of biosynthesis by the wild strain A-101, its acetate-negative mutant Wratislavia 1.31 and transformant strains, obtained in bioreactor cultures with glycerol medium. For abbreviations see the Abbreviations section of this document.

However, a high rate of glycerol utilization was not linked with high erythritol secretion in the medium culture of both of the aforementioned strains was characterized by the lowest erythritol concentrations (Figure 5A). However, these strains stood out in terms of mannitol production, which was to about 20 g/L.

Mannitol biosynthesis often accompanies the biosynthesis of erythritol and citrate by *Y. lipolytica*, but it is usually utilized by the yeast even before glycerol is depleted. This usually leads to lowering of the final concentration of mannitol in the post-culture broth to several grams per liter. In addition, it is known that in *Y. lipolytica* cells, high osmotic pressure primarily promotes the formation of erythritol rather than mannitol. Moreover, addition of NaCl to the medium additionally inhibits mannitol dehydrogenase, favoring pure erythritol biosynthesis [54].

It is worth emphasizing that the strains 1.31.GUT1/6 and 1.31.GUT1/6.CIT1/3 consumed glycerol very quickly (Figure 4), thus lowering the osmotic pressure, which in turn could correlate with a higher production of mannitol. Furthermore, no significant differences in biomass levels were observed between these strains, so rapid input of the carbon source into the cell could be easily directed to gluconeogenesis and to the pentose phosphate pathway. An intermediate compound in the pathway of glycerol to glucose-6-phosphate is fructose-1,6-bisphosphate and then fructose-6-phosphate, which is a direct precursor of mannitol-1-phosphate, which may explain the high concentration of mannitol in the cultures of these strains.

Strains containing the organic acid transporter gene, 1.31.GUT1/6.CIT1/3.E34672g and 1.31.E34672g, were characterized by very high secretion of total metabolites, 103.5 and 114.0 g/L, respectively (Figure 5A). Among these products, polyols—erythritol and mannitol—predominated; however, for the strain 1.31.E34672g, formation of a relatively high amount (34.7 g/L) of citrates was observed. In this study, two transformants of the Wratislavia 1.31 strain, 1.31.GUT1/6.CIT1/3.E34672g and 1.31CIT1/3, produced 12% and 3% more erythritol with a higher yield than the parental strain. It was evident that in the 1.31.GUT1/6.CIT1/3.E34672g strain, the introduction of the E34672g gene significantly improved the erythritol biosynthesis process compared to the 1.31.GUT1/6.CIT1/3 strain. It seems that the increased flux of intracellular metabolites between the mitochondrion and cytoplasm proved to be crucial for this process. In the media for erythritol and citrates production used in this study, the C:N ratio was 110:1, so the limiting factor for yeast growth in both cases was a low nitrogen level.

Depletion of nitrogen from the environment causes an increase in the production of citrates in the mitochondria, which, after exceeding a certain level, are secreted into the cytoplasm. However, the process occurs under conditions of increased osmotic pressure, against which the cell defends itself by synthesizing polyols, which will eventually be secreted and re-used as an additional pool of carbon sources, and this may result in the accumulation of larger amounts of organic acids in the mitochondria. This theory is confirmed by the fact that in the culture of the 1.31.GUT1/6.CIT1/3.E34672g strain a slow increase of biomass was observed until the end of the process.

As mentioned above, the erythritol production from glycerol includes substrate assimilation followed by reactions of the pentose phosphate pathway. Dihydroxyacetone phosphate created by the action of glycerol kinase and glycerol-3-P dehydrogenase is converted by triosephosphate isomerase into glyceraldehyde-3-phosphate which, after gluconeogenesis, enters the pentose phosphate pathway. The gene encoding triosephosphate isomerase (*TPI1*; *YALIOF0521g*) was used to construct strain FCY207, which showed an approximately 5% increase in erythritol production [55]. However, among the enzymes of the pentose phosphate pathway transketolase is of particular importance in this process [55,56]. In the case of the FCY208 strain, overexpressing the *TKL1* gene (encoding transketolase) the erythritol production efficiency was 0.59 g/g compared to 0.46 g/g for the wild strain JMY2900WT [55]. The AMMpAD-TKL1 transformant produced erythritol with a yield of 0.58 g/g, whereas the parental strain MK1 produced erythritol with a yield of 0.54 g/g under the same conditions [56] (Table 4).

However, overexpression of the second enzyme from the non-oxidative phase of the pentose phosphate pathway—transaldolase—did not bring about any significant changes in the amount and rate of erythritol production [56]. Created by the action of transketolase and/or transaldolase, erythrose-4-phosphate is dephosphorylated by erythrose-4-phosphatase to erythrose, which is then reduced to erythritol by erythrose reductase. According to Carly et al. [55], overexpression of the latter two enzymes does not improve erythritol production. However, Janek et al. [58] found that overexpression of the gene *YALI0F18590g*, encoding erythrose reductase from *Y. lipolytica,* enhanced the titer of erythritol 20% over the control (44.44 g/L). Significantly better titers than those described above were obtained as a result of the synergistic effect of simultaneous overexpression of two or three genes. Co-expression of *GUT1* along with *TKL1* or *GUT1* with *ALR* had a significant effect on the increase of erythritol production [55]. Similarly, in the present study, the best parameters of erythritol production were obtained as a result of co-overexpression of three genes. It is also worth emphasizing that the strain that produced the highest amount of erythritol in our current study was also reported to be capable of producing large quantities of α-ketoglutaric acid [46].

## 3. Materials and Methods

### 3.1. Microorganisms

The wild strain of *Y. lipolytica* A-101 and Wratislavia 1.31, an acetate-negative UV mutant obtained in the A-101 strain [53], as well as five transformants derived in Wratislavia 1.31 obtained using methods described by Tomaszewska et al. [46] were used in this study. Briefly, the genes *GUT1* (glycerol kinase), *CIT1* (methylcitrate synthase) and *YALI0E34672g*, encoding the mitochondrial succinate-fumarate transporter, were overexpressed (separately or in combinations). The expression of these genes was controlled by strong, constitutive *TEF* promoter, and integration into the genome was achieved using ZETA sequences (retrotransposon sequences), which allows for random insertion.

### 3.2. Media

The medium for inoculum preparation for citric acid biosynthesis had the following composition [g/L]: glycerol—50; NH_4_Cl—1; KH_2_PO_4_—0.2; MgSO_4_×7H_2_O—1; yeast extract—1. The growth medium for erythritol biosynthesis in 1 L contained: glycerol 50.0 g; yeast extract 3.0 g; malt extract 3.0 g; Bacto Peptone 5.0 g. Spark Tecan microculture mineral medium contained in g/L: glycerol—20; NH_4_Cl—9.6; KH_2_PO_4_—2.0; MgSO_4_×7H_2_O—1.4 and thiamine—3 µg/L. Substrates for morphological evaluation of colonies were: YNB_t_, YNB_YE_ and YM. The YNB media used 0.67 g/L YNB (Yeast Nitrogen Base without vitamins—Sigma-Aldrich, Steinheim, Germany), 20 g/L glycerol and 20 g/L agar, as well as 6 µg/L of thiamine (YNB_t_) or 0.1 g/L of yeast extract (YNB_YE_). The YM medium in 1 L contained: glycerol 20.0 g; yeast extract 3.0 g; malt extract 3.0 g; Bacto Peptone 5.0 g; agar 20.0 g. Shake flask culture medium (for citric acid biosynthesis) contained [g/L]: glycerol—100; NH_4_Cl—1; KH_2_PO_4_—0.2; MgSO_4_·7H_2_O—1; yeast extract—1. The pH of the medium was stabilized by the addition of 5 g/L CaCO_3_. The production medium for citric acid biosynthesis in a 1 L bioreactor contained: glycerol—150 g; NH_4_Cl—2 g; KH_2_PO_4_—0.2 g; MgSO_4_·7H_2_O—1 g; yeast extract—1 g, and for erythritol production an additional 25 g of NaCl. Media for shaking cultures in the Spark Tecan apparatus and for agar plates were prepared in distilled water, whereas for bioreactor cultures tap water was used. All chemicals used in the study were of analytical purity (Sigma-Aldrich, Steinheim, Germany).

### 3.3. Culture Conditions

The growth culture was carried out in 0.3-L flasks containing 0.05 L of growth medium on a rotary shaker (CERTOMAT IS; Sartorius, Goettingen, Germany) at 29.0 °C and 140 rpm for 72 h. Citrate production in shake flask cultures was carried out in baffled flasks containing 0.03 L of medium under conditions described above for 7 days. The cultures were performed in triplicate.

### 3.4. Microcultures

Inoculum cultures of transformants prepared in mineral medium were centrifuged (Eppendorf Centrifuge 5810R; Hamburg, Germany) for 10 min at 10,000 rpm. Subsequently, the supernatant was discarded, and the pellet of yeast biomass was suspended in fresh medium of the same composition. The optical density was set at 2 on the McFarland scale (OD_600_ of about 0.5). Next, cell suspensions were transferred to a 96-well microplate. As a control, the uninoculated medium was introduced to the plate. The microcultures were carried out in triplicate in a Spark Tecan device (Männedorf, Switzerland) for 72 h at 28 °C. The growth curves were plotted based on OD_600_ measurements collected by the device every 30 min.

### 3.5. Bioreactors Studies

The cultivations for citrate and erythritol production were performed in a Biostat B Plus 5-L bioreactor (Sartorius, Melsungen, Germany) with a working volume of 2 L at 29.5 °C. The stirrer rate was set to 800 rpm and the aeration was fixed at 0.8 L/min. The pH was maintained automatically at 5.5 (citrate production) or 3.0 (erythritol biosynthesis) via the addition of NaOH (20 or 40%, respectively). The cultures were performed in two biological replicates.

### 3.6. Analytical Methods

Culture samples (10 mL) were collected after the end of shake-flasks cultures and every 24 h from bioreactor cultures, centrifuged (10 min, 5000 rpm) and filtered on a membrane filter with a pore size of 0.45 μm. The biomass was dried on a weighing dryer (RADWAG MAC 110/NH, Radom, Poland) at 105 °C to constant weight. The supernatant was used for HPLC analysis according to the method described earlier [39]. The concentration of isocitric acid in the supernatant was determined using an Isocitrate Assay Kit (Sigma-Aldrich) according to the methodology provided by the manufacturer. Statistical analysis was done using one-way analysis of variance (Statistica 13.0 software; StatSoft, Tulsa, OK, USA). Significant differences in the data were detected by Duncan’s multiple range test (*p* ≤ 0.05).

## 4. Conclusions

To summarize the results of this study, after genetic engineering of the acetate-negative mutant Wratislavia 1.31, transformants with improved ability to produce these metabolites were obtained. The modifications assumed improvement of glycerol utilization (overexpression of the *GUT1* gene), improvement of the Krebs cycle performance (*CIT1*) and secretion of organic acids from the mitochondria through overexpression of the *YALI0E34672g* gene, and the assessment of the synergistic effect of overexpression of a combination of these genes. Among the analyzed strains, the 1.31.GUT1/6 and 1.31.GUT1/6.CIT1/3 strains were characterized by a high rate of glycerol utilization, both under conditions favoring citrate biosynthesis (pH 5.5) and those favoring erythritol formation (pH 3.0 and high osmotic pressure). However, the analyzed modifications did not guarantee the selective production of citric acid or erythritol. In the case of both transformants the obtained post-culture broth was characterized by a higher content of citric acid compared to the parental strain Wratislavia 1.31, at the same time containing a comparatively large amount of isocitrate in the total pool of citrates and erythritol. The flexibility and metabolic potential of *Y. lipolytica* make it possible to simultaneously overproduce significant amounts of several compounds in one process. On the one hand, this reduces the purity of the main metabolite, but on the other hand, it allows a mixture of several high-value products to be obtained. Therefore, determining a strain with high production potential to biosynthesize more than one metabolite may lead to obtaining a new product for which a use should be found. In this study, in the pool of products produced in the process of erythritol biosynthesis by the above-mentioned strains, 22% of the metabolites were represented by mannitol—another valuable compound with similar properties to erythritol; therefore, it does not necessarily have to be eliminated from the post-culture broth, which might significantly reduce purification costs and increase the profitability of the production process.

In the present study the greatest improvement in erythritol production parameters was observed for *Y. lipolytica* 1.31.GUT1/6.CIT1/3.E34672g, overexpressing all three examined genes. The strain produced 84 g/L of erythritol with a yield and productivity of 0.54 g/g and 0.72 g/Lh, respectively, which places this transformant strain in the group of the currently highest-ranked *Y. lipolytica* erythritol producers. Considering the satisfactory results obtained by simultaneous overexpression of various genes in this study, future research should focus on implementing co-overexpression of further genes regulating different steps of these pathways.

## Figures and Tables

**Table 1:** Growth parameters of *Y. lipolytica* strains growing in Spark Tecan apparatus in mineral medium with thiamine deficiency.

No.	Strain	OD_600_	µ_max_ [h^−1^]
1	A-101	0.45	0.100
2	Wratislavia 1.31	0.25	0.081
3	1.31.GUT1/6	0.37	0.065
4	1.31.GUT1/6.CIT1/3	0.42	0.080
5	1.31.GUT1/6.CIT1/3.E34672	0.48	0.087
6	1.31.E34672g	0.42	0.094
7	1.31.CIT1/3	0.42	0.097

For abbreviations see the Abbreviations section of this document.

**Table 2:** Citrate biosynthesis by mutants and genetically modified strains of Y. lipolytica—summary of results from the last decade.

Strain of *Y. lipolytica*	Substrate	Culture Mode	CA + ICA [g/L]	CA [g/L]	CA	Q_CA+ICA_ [g/Lh]	Q_CA_ [g/Lh]	Y_CA+ICA_ [g/g]	Y_CA_ [g/g]	References
NCYC3825	glycerol	fed-batch	58.8	-	no data	0.86	-	0.17	-	[47]
AJDpADUTGut1	glycerol	batch	60.4	-	no data	0.63	-	0.40	-	[43]
K1 INU 6	fructose	fed-batch	124.0	94.0	75.8	0.53	0.40	0.62	0.48	[48]
	inulin		120.2	105.2	87.5	0.51	0.44	0.60	0.53	
PG86	glucose	fed-batch	101.0	-	no data	0.42	-	0.89	-	[49]
PR32	glucose	fed-batch	111.1	-	no data	0.46	-	0.93	-	[50]
XYL+	xylose	batch	79.4	-	no data	0.53	-	0.91	-	[5]
AJDpADUT-Gut1/2	glycerol	batch	75.9	42.5	56.0	0.53	0.23	0.50	0.22	[51]
AWG7	fructose	batch	50.7	48.7	96.0	0.44	0.42	0.50	0.49	[39]
AWG 7 INU 8	inulin		77.0	75.5	98.0	0.82	0.80	0.77	0.76	
1.31.CIT2	glycerol	batch	92.8	72.1	77.7	0.64	0.53	0.57	0.43	[44]
GUT.1/6	glycerol	batch	87.5	60.4	69.0	0.77	0.53	0.60	0.41	This work

For abbreviations see the Abbreviations section of this document.

**Table 3:** Characterization of citrate biosynthesis by wild-type strain A-101, its acetate-negative mutant Wratislavia 1.31, and the transformants of *Y. lipolytica* Wratislavia 1.31 in bioreactor cultures using glycerol as the only carbon source.

Strain	CA	ICA	CA + ICA	CA/ (CA + ICA)	ERY	MAN	Q_CA+ICA_	Q_CA_	Y_CA+ICA_	Y_CA_	q_CA+ICA_	q_CA_
	[g/L]			(%)	[g/L]		[g/Lh]		[g/g]		[g/gh]	
1.31.GUT.1/6	60.4	27.1	87.5	69.0	24.2	0.0	0.77	0.53	0.60	0.41	0.066	0.045
1.31.GUT1/6.CIT1/3	53.3	27.7	81.0	65.8	28.7	7.1	0.91	0.60	0.53	0.35	0.063	0.041
A-101	66.5	17.8	84.3	70.6	3.4	4.9	0.83	0.66	0.59	0.47	0.058	0.046
Wratislavia 1.31	73.3	3.2	76.5	95.8	4.8	2.9	0.79	0.76	0.50	0.48	0.057	0.055

For abbreviations see the Abbreviations section of this document.

**Table 4:** Comparison of erythritol production by *Y. lipolytica* wild-type strain A-101, its acetate-negative mutant Wratislavia 1.31 and genetically engineered transformants in bioreactor cultures with glycerol.

Strain of *Y. lipolytica*	Improvement Strategy	Culture System; Production Volume	Carbon Source [g/L]	ERY [g/L]	Y_ERY_ [g/g]	Q_ERY_ [g/Lh]	Ref.
M53	Mutagenesis ARTP	Bioreactor batch culture 3 L	Waste cooking oil—30	22.1	0.74	0.32	[57]
Wratislavia K1	Isolation from the acetate-negative mutant Wratislavia 1.31 strain in the course of continuous citric acid production from glucose in a nitrogen-limited chemostat culture at a dilution rate of D = 0.016 h^−1^	Bioreactor batch culture; 2 L	Pure glycerol—150	84.1	0.50	0.86	[13]
			Crude glycerol—150	80.0	0.49	1.00	
AMM pAD-YlER	Overexpression of *YALI0F18590g* gene encoding predicted erythrose reductase in UV mutant (MK1 strain)	Bioreactor batch culture; 2 L	Pure glycerol—150	79.0	0.53	1.00	[58]
AJD pADUTGut1	Overexpression of *YALI0F00484g* gene (*GUT1*) encoding glycerol kinase and gene *YALI0B02948g* (*GUT2*) encoding glycerol-3-P dehydrogenase	Bioreactor batch culture; 2 L	Pure glycerol—150	71.3	0.48	0.99	[43]
AJD pADUTGut2				42.0	0.28	0.58	
AJD pADUTGut1/2				78.0	0.52	1.08	
MK1	UV mutagenesis of Wratislavia K1 strain	Bioreactor batch culture 2 L	Pure glycerol—150	82.2	0.55	0.84	[59]
MK1	Overexpression of *YALI0B15598g* encoding 6-phosphogluconate dehydrogenase (*GND1*); *YALI0E22649g*—glucose-6-phosphate dehydrogenase (*ZWF1*), *YALIOE06479g*—transketolase (*TLK1*) and *YALIOF15587g*—transaldolase (*TAL1*)	Baffled flask; 25 mL	Pure glycerol—100	54.15	0.54	0.75	[56]
AMM pAD-GND1				50.67	0.51	0.70	
AMM pAD-ZWF1				50.59	0.51	0.70	
AMM pAD-TLK1				57.99	0.58	0.81	
AMM pAD-TAL1				43.13	0.45	0.63	
FCY218	Overexpression of *GUT1* and *TKL1* and disruption of EYK1 in transformant Po1d (ura3-302 leu2-270 xpr2-322)	Bioreactor batch culture; 1 L	Pure glycerol—150	80.6	0.53	1.03	[55]
AJD45,13 pAD-VHb	Overexpression of codon-optimized bacterial hemoglobin (VHb) from *Vitreoscilla stercoraria*	Bioreactor batch culture; 2 L	Pure glycerol—150	55.75	0.37	0.38	[60]
Wratislavia K1	The inulinase gene (*INU1* gene; X57202.1) amplified from *Kluyveromyces marxianus* CBS6432 was cloned into the genome of *Y. lipolytica* Wratislavia K1 strain	Bioreactor batch culture; 2 L	Glycerol—40 + 200	95.0	0.48	1.28	[48]
K1 INU6				120.9	0.60	1.23	
Wratislavia 1.31	Overexpression of *YALI0F00484g* gene (*GUT1*) encoding glycerol kinase, *YALI0E00638g*—methylcitrate synthase and mitochondrial transporter *YALI0E34672g* in Wratislavia 1.31 strain	Bioreactor batch culture; 2 L	Pure glycerol—150	74.5	0.5	0.67	This study
GUT.1/6.CIT.1/3.E34672				84.0	0.54	0.72	

For abbreviations see the Abbreviations section of this document.

## Data Availability

Data is contained within the article.

## Abbreviations

CA	citric acid
ERY	erythritol
GLY	glycerol
ICA	isocitric acid
MAN	mannitol
µ_max_	maximal specific growth rate in logarithmic phase
OD	optical density measured at λ = 600 nm
X	biomass
Q_GLY_	volumetric consumption rate of glycerol
Q_CA_, Q_ERY_	volumetric production rates of citrates (CA) and erythritol (ERY)
q_GLY_	specific consumption rate of glycerol
q_CA_, q_ERY_	specific production rates of citrates (CA) and erythritol (ERY)
Y_CA_, Y_ERY_	yield of citrates (CA) and erythritol (ERY) (g of product per g of used substrate)
